# MR imaging findings of massive perivillous fibrin deposition of the placenta: A case report

**DOI:** 10.1016/j.radcr.2024.03.090

**Published:** 2024-04-24

**Authors:** Shinya Fujii, Naoko Mukuda, Hiroto Yunaga, Takuro Gonda, Takeru Fukunaga, Yuji Kamata, Ryoya Ochiai, Kanae Ozaki

**Affiliations:** a Division of Radiology, Department of Multidisciplinary Internal Medicine, Faculty of Medicine, Tottori University, Yonago, Japan; b Department of Pathology, Faculty of Medicine, Tottori University, Yonago, Japan

**Keywords:** Magnetic resonance imaging, Massive perivillous fibrin deposition, Placenta, Uterine

## Abstract

Massive perivillous fibrin deposition (MPFD) of the placenta is characterized by the obliteration of the villous trophoblast with extensive deposition of fibrinoid material in the intervillous space. Here, we describe the MRI findings of a case of MPFD. The placenta demonstrates linear and geographical hypointensity on T2-weighted imaging, which is suggested to mainly reflect fibrin deposition. This finding should be noted, particularly in patients with miscarriage in their past history.

## Introduction

Massive perivillous fibrin deposition (MPFD) of the placenta is characterized by the obliteration of the villous trophoblast with extensive deposition of fibrinoid material in the intervillous space, which leads to functional failure of the placenta extending over at least 25 % of the placental volume [1,2]. MPFD is a rare condition associated with serious adverse pregnancy outcomes including spontaneous abortion, intrauterine growth restriction (IUGR), and fetal death. Autoimmunity, infection, thrombophilia, and genetic predisposition has been proposed as the etiology [2,3]. However, the precise mechanisms of MPFD are still unknown. Magnetic resonance imaging (MRI) findings of MPFD are not well known. Herein, we report a case of recurrent MPFD on MRI.

## Case report

A 39-year-old pregnant woman (gravida 2, para 1) with intrauterine growth restriction (IUGR) and a sign of threatened preterm labor was referred to our hospital at 35 weeks of gestation, although she had been treated with low-dose aspirin from early gestation. On her previous gestation, pregnancy-induced hypertension, severe IUGR, and neonatal death occurred. Fibrin deposition in the placenta was revealed on pathological examination after the previous pregnancy.

**Fig. 1:**
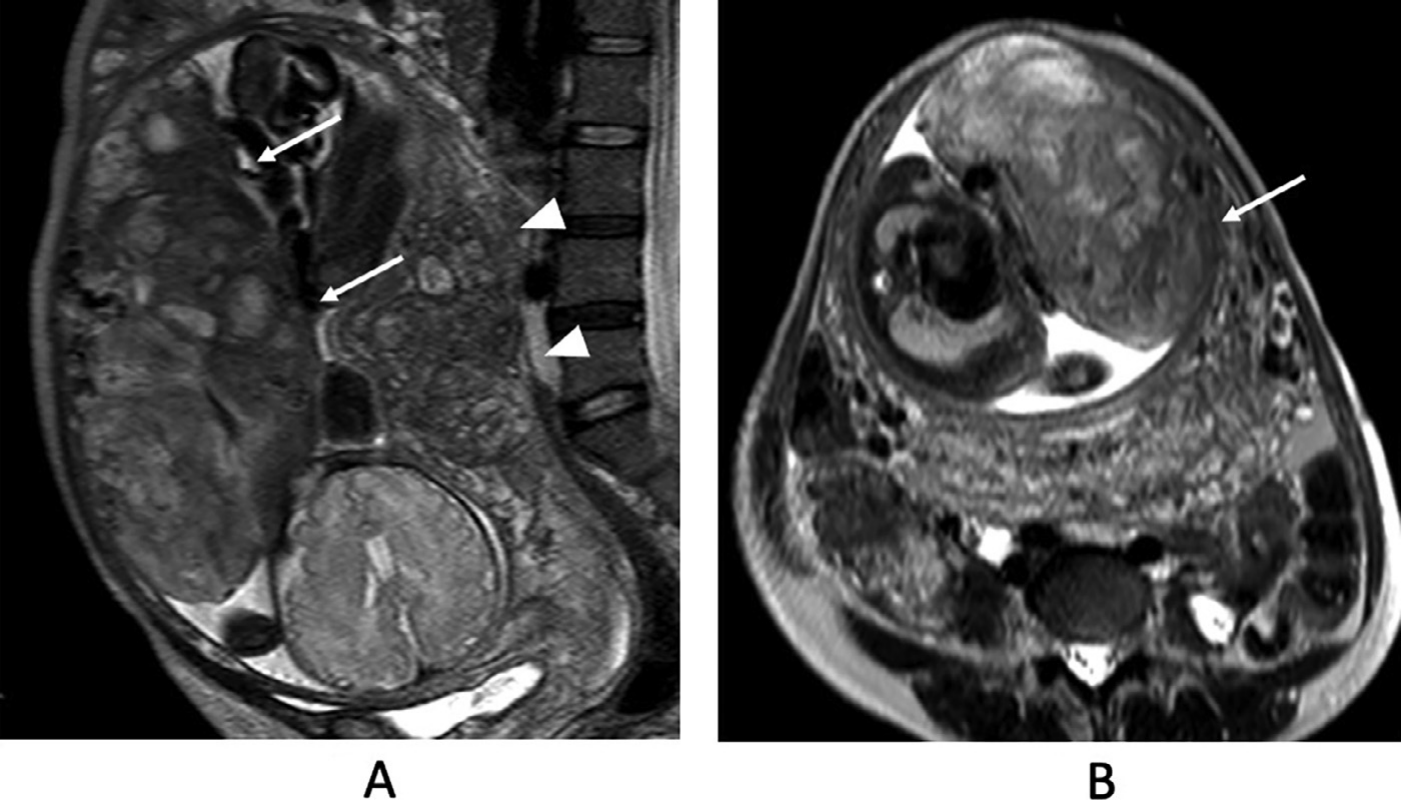
Single-shot turbo spin-echo images (T2-weighted images). (A and B) Magnetic resonance imaging (MRI) obtained at 36 weeks of gestation during the reported pregnancy. T2-weighted images reveal oligohydramnios and a thickened placenta. The placenta shows heterogeneous signal intensity with abnormal intraplacental linear and geographical hypointensity on T2-weighted images (A, B arrows). Adenomyosis is also observed in the posterior uterine wall (A, B arrowheads).

**Fig. 2:**
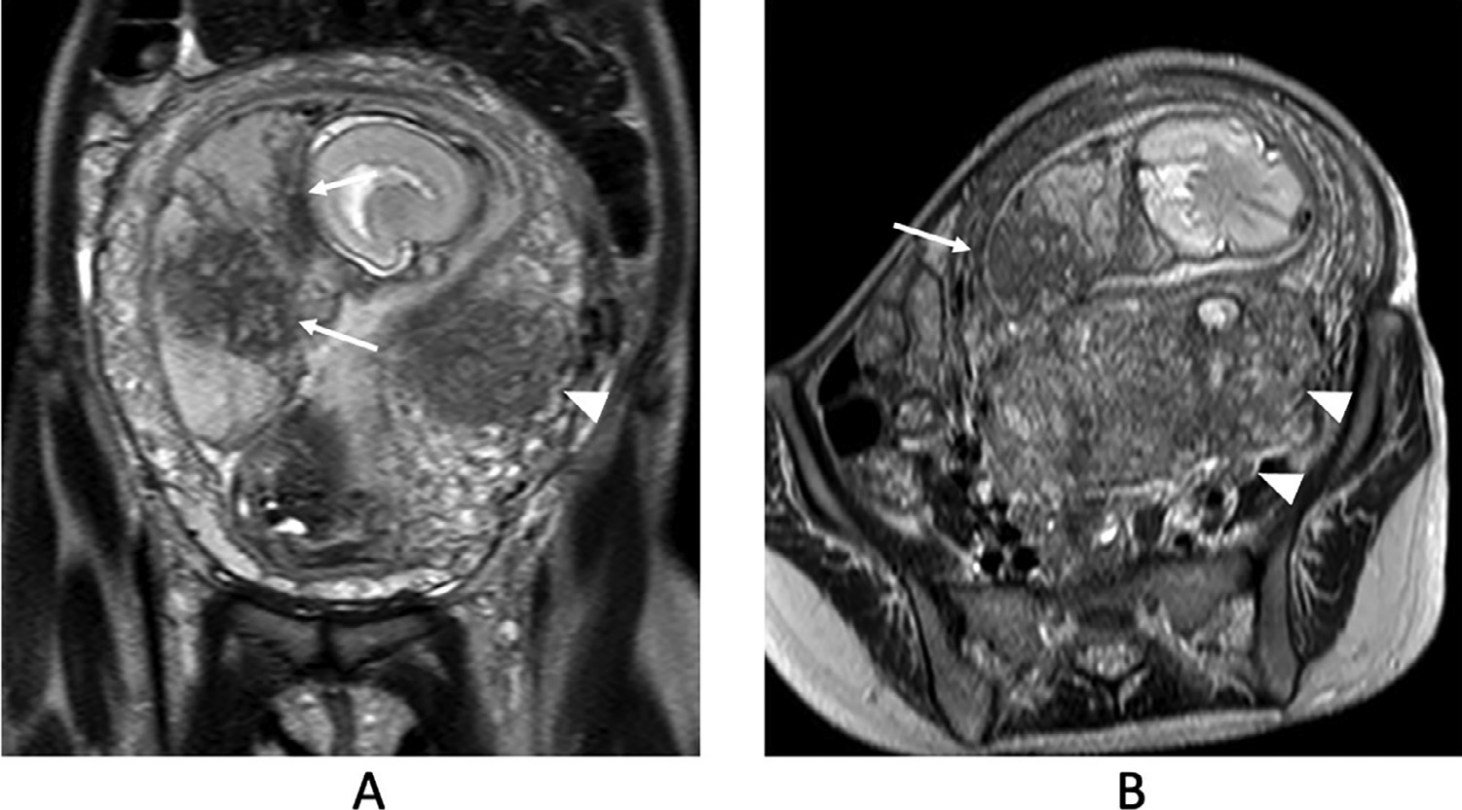
Single-shot turbo spin-echo images (T2-weighted images). (A and B) MRI obtained at 27 weeks of gestation during the previous pregnancy. The T2-weighted images obtained at 27 weeks of gestation at the previous pregnancy also demonstrate the same findings, such as intraplacental linear or geographical hypointensity (arrows) with oligohydramnios. Adenomyosis is also observed (A arrowheads).

**Fig. 3:**
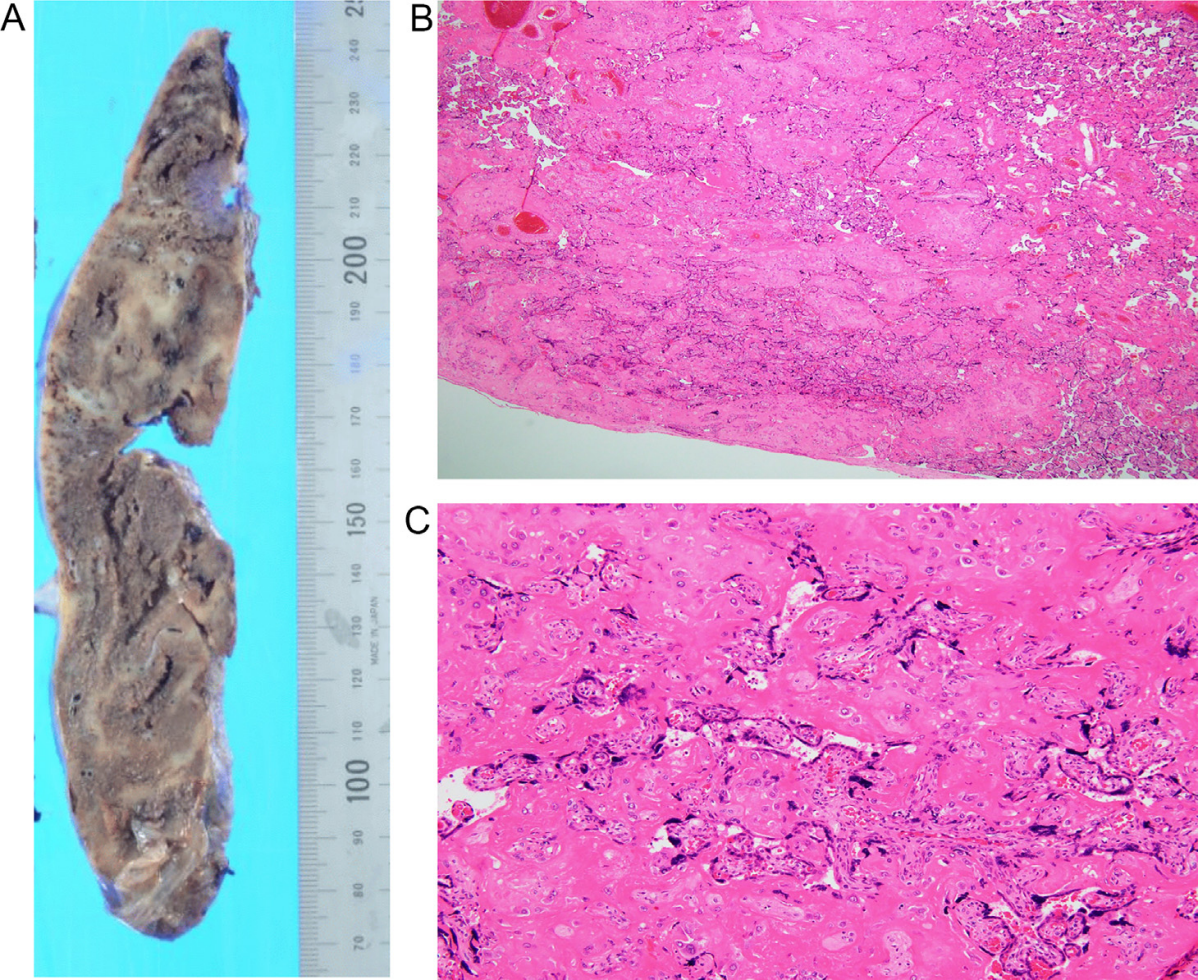
(A) gross specimen, (B) hematoxylin and eosin (H & E) stain low-power field (×20), (C) H & E stain high-power field (×200). On gross examination, the placenta is thickened, with a smaller diameter than that considered normal as per the patient's gestational weeks. Mild to moderate fibrin deposition is noted, and lattice-like fibrin deposition from the decidua to the chorionic plate is found in over 50% of cutting surfaces (A, grayish white). Microscopically, diffuse fibrin deposition is observed and multiple intervillous thrombus formation are also noted (B). Fibrin deposition is mainly perivillous. Although the villi are rather atrophic and their capillaries tend to collapse, there is no necrotic villus (C).

An ultrasound examination performed at 36 weeks of gestation demonstrated oligohydramnios (amniotic fluid index: AFI = 6 cm) without clinical evidence of membrane rupture. MRI was performed at 36 weeks of gestation (Fig. 1). MRI revealed oligohydramnios and a thickened placenta. The placenta showed heterogeneous signal intensity with abnormal intraplacental linear and geographical hypointensity on single-shot turbo spin echo images (T2-weighted images) (Fig. 1). No abnormal signal intensity was observed on T1-weighted images. At that time, the MRI obtained at 27 weeks of gestation during the previous pregnancy was retrospectively evaluated. The placenta demonstrated similar findings, such as intraplacental linear and geographical hypointensity on T2-weighted images (Fig. 2). Emergency cesarean section was performed because severe abdominal pain occurred 3 days after admission. On gross examination, the placenta was thickened, with a smaller diameter than that considered normal for the patient's gestational age. Mild to moderate fibrin deposition was noted, and lattice-like fibrin deposition from the decidua to the chorionic plate was found over 50% of the cutting surfaces. Microscopically, diffuse fibrin deposition was observed and multiple intervillous thrombus formations were also noted. Fibrin deposition was mainly perivillous. Although the villi were rather atrophic and their capillaries tended to collapse, there was no necrotic villus (Fig. 3). The patient was pathologically diagnosed with MPFD.

## Discussion

The estimated incidence of MPFD is generally difficult because of the lack of routine placenta examinations. However, the incidence after 22 weeks of gestation was at 1.1 % and higher to 2.7 % in recurrent early miscarriages [2]. Some etiologies including infection have been proposed. Moreover, MPFD associated with SARS-CoV-2 infection has been recently reported [2].

In the present case, the placenta demonstrated linear and geographical hypointensity on T2-weighted imaging, which was suggested to reflect fibrin deposition. In an article on MRI findings of placental diseases by Linduska et al. [4], MPFD could not be detected by MRI in any of the six cases that were included in the article. They described that the fibrin deposition showed iso-intensity on all sequences, including T2-weighted imaging. However, dark intraplacental bands on T2-weighted imaging, which are important for the diagnosis of placenta accreta, are thought to represent areas of fibrin deposition within the placenta [5]. Additionally, a recent report about placental MRI of perivillous fibrin deposits due to SARS-CoV-2 infection demonstrated that the placenta showed heterogeneous T2 hypointensity most likely corresponding to the fibrin deposits [6]. Therefore, we believe that the low intensity observed in this present case contributes to intraplacental fibrin deposition. A clear explanation regarding the discrepancy between the previous report [4] and the present case is difficult. However, a possible reason causing the discrepancies could be the different degrees of fibrin deposition in individual cases. According to the criteria reported by Katzman and Genest [1], MPFD is classified into two types: transmural and borderline MPFD. Transmural MPFD is defined as perivillous fibrinoid material extending from the maternal surface to the fetal surface, encasing ≥50% of the villi on at least 1 histological slide. On the other hand, borderline MPFD is defined as 25%-50% villi on at least one slide encased by perivillous fibrinoid material in a transmural or a nearly transmural distribution [1]. Consequently, the amount of fibrin might not be enough to be detected on MRI in the cases of borderline MPFD.

Himoto et al. reported that the presence of intraplacental diffuse regular hypointensity on T2-weighted imaging, a black-and-white 2-tone pattern, is significantly associated with the presence of pathologically proven placental insufficiency [7]. This 2-tone pattern is suggested to reflect compensatory alternations like the distribution of villus, maternal blood, and oxygen saturation, in the respective cotyledons caused by placental hypo-circulation [8]. In their study [7], various conditions such as excessive fibrin deposition, very small placenta of less than the tenth percentile, placental villous hypermaturation, and chorangiosis were included as placental insufficiency. Therefore, placental hypo-circulation might contribute to placental hypointensity in the present case.

In this present case, placental heterogeneous T2 hypointensity was found in this pregnancy as well as in the previous pregnancy. The risk of recurrence in MPFD has been reported to range from 12% to 80% [2,3]. Therefore, MPFD should be considered in the case with similar placental heterogeneous T2 hypointensity in the previous pregnancy.

In conclusion, we described the MRI findings of a case of MPFD. The placenta demonstrates linear and geographical hypointensity on T2-weighted imaging, which is suggested to mainly reflect fibrin deposition. This finding should be noted, particularly in patients with miscarriage in their past history.

## Patient consent

Written informed consent was obtained from the patient prior to submission of this case report.
